# Genome-wide analysis of regions similar to promoters of histone genes

**DOI:** 10.1186/1752-0509-4-S1-S4

**Published:** 2010-05-28

**Authors:** Rajesh Chowdhary, Vladimir B Bajic, Difeng Dong, Limsoon Wong, Jun S Liu

**Affiliations:** 1 Department of Statistics, Harvard University, Cambridge, MA 02138, USA; 2 Biomedical Informatics Research Center, MCRF, Marshfield Clinic, 1000 North Oak Avenue, Marshfield, WI 54449, USA; 3 Computational Bioscience Research Center (CBRC), King Abdullah University of Science and Technology (KAUST), Thuwal 23955-6900, Kingdom of Saudi Arabia; 4 School of Computing, National University of Singapore, Singapore 117417

## Abstract

**Background:**

The purpose of this study is to: i) develop a computational model of promoters of human histone-encoding genes (shortly histone genes), an important class of genes that participate in various critical cellular processes, ii) use the model so developed to identify regions across the human genome that have similar structure as promoters of histone genes; such regions could represent potential genomic regulatory regions, e.g. promoters, of genes that may be coregulated with histone genes, and iii/ identify in this way genes that have high likelihood of being coregulated with the histone genes.

**Results:**

We successfully developed a histone promoter model using a comprehensive collection of histone genes. Based on leave-one-out cross-validation test, the model produced good prediction accuracy (94.1% sensitivity, 92.6% specificity, and 92.8% positive predictive value). We used this model to predict across the genome a number of genes that shared similar promoter structures with the histone gene promoters. We thus hypothesize that these predicted genes could be coregulated with histone genes. This hypothesis matches well with the available gene expression, gene ontology, and pathways data. Jointly with promoters of the above-mentioned genes, we found a large number of intergenic regions with similar structure as histone promoters.

**Conclusions:**

This study represents one of the most comprehensive computational analyses conducted thus far on a genome-wide scale of promoters of human histone genes. Our analysis suggests a number of other human genes that share a high similarity of promoter structure with the histone genes and thus are highly likely to be coregulated, and consequently coexpressed, with the histone genes. We also found that there are a large number of intergenic regions across the genome with their structures similar to promoters of histone genes. These regions may be promoters of yet unidentified genes, or may represent remote control regions that participate in regulation of histone and histone-coregulated gene transcription initiation. While these hypotheses still remain to be verified, we believe that these form a useful resource for researchers to further explore regulation of human histone genes and human genome. It is worthwhile to note that the regulatory regions of the human genome remain largely un-annotated even today and this study is an attempt to supplement our understanding of histone regulatory regions.

## Background

Gene regulation represents a complex process that determines which genes would express in a particular cell, at a particular time, and by how much. Such *suited to purpose* gene regulation programs are essential for normal functioning of cells in all living organisms. In order to understand these biologically important mechanisms of gene regulation, it is crucial to unravel genes that are likely coregulated with each other. Such genes need not have the same function, but they generally coordinate their activities to provide proper cell response on different conditions and stimuli. In this study we focus on the histone-encoding gene (histone genes, henceforth) coregulation problem. Histones have been recognized to play a crucial role in various cellular functions related to DNA packaging into nucleosomes, chromatin composition, and gene transcription and regulation [1]. Using a computational methodology we have conducted a genome-wide study with the aim to discover regions that have similar structures as promoters of histone genes. Our assumption is that two genes with sufficiently similar promoter structures have an increased chance to be coregulated and consequently coexpressed. This stems from the understanding that one of the key point of gene regulation happens at the transcription stage where the promoters play the most crucial role. To determine these potential regulatory regions, we developed a statistical model to capture regulatory signals in the promoters of human histone genes, which we then used to analyze the human genome. This was done using Dragon Promoter Mapper (DPM) tool, a Bayesian network based framework we developed earlier [2] for modeling promoter of specific classes of genes that may be coregulated. The resulting histone promoter model showed an excellent performance in leave-one-out cross-validation tests (94.1% sensitivity, 92.6% specificity, and 92.8% positive predictive value (ppv)). When applied the tool on a genome-wide scale, we were able to not only recover correctly the majority of the histone genes used for training, but also predict a large number of other genes that share similar promoter structures with histone genes and we thus believe that they are likely to be coregulated and thus coexpressed with the histone genes. These results matched well with the known experimental data and we found them to be statistically significant.

To the best of our knowledge this is the most comprehensive computational analysis conducted thus far on a genome-wide scale where genes coregulated with the histone genes were searched for based on the promoter model derived from an extensive collection of histone genes. Previous studies in this area have mostly been conducted on either single histone genes [1,3-16] or a handful of them [17-19]. There have been studies of similar nature in the past that have been conducted on other gene types, though they are very few probably due to unavailability of sufficient, relevant and clean data. Some of these include study done on muscle specific genes by Wasserman et al [20].

## Methods

We used the standalone version of DPM tool [2] to develop a Bayesian network (BN) based histone promoter model by exploiting several features that may reflect the biology associated with these promoters. These features include putative transcription factor binding sites (TFBSs) present in the promoters, their order of occurrences, their locations, and the mutual distances among them. These features essentially encode the modular arrangement of the binding sites in the promoters which could be critical to transcription regulation functions, as having been shown earlier [17-23]

### Data collected

Using Entrez Gene (http://www.ncbi.nlm.nih.gov/entrez/query.fcgi?db=gene), we identified 86 human histone genes whose information was available in the database. Of these 86 genes we randomly selected 68 (79%) for training our histone promoter model. This was done in order to see if our model was able to predict the left-out genes during the whole genome scan analysis. Using the UCSC Genome Browser (http://genome.ucsc.edu), we then collected promoter segments of the 68 training histone genes covering a region of [-500,+100] with respect to the TSS (refer to Additional file 1 for the collected sequences), which was based on their most 5’ transcript. This region around the TSS is known to contain a large majority of binding sites that regulate histone genes [3,17-19]. We also collected 68 background sequences of the same length, selected randomly from the human genome. Collectively, these 136 promoter and background sequences formed the training sequences which were used to train our model. We used the balanced number of promoter and background sequences in our training set to eliminate the effect of class bias in the model prediction. In order to see the effect of bias of background sequence selection on the model performance, we analyzed the training sequence data containing 10 separate background sequence sets collected randomly from the genome. Using UCSC Genome Browser, we also collected for further analysis 25 human chromosomal sequences (build HG18), Chr1 through Chr22, ChrM, ChrX and ChrY.

In addition, we collected 10 position weight matrices (PWMs) of binding boxes and TFBSs known to be present in histone promoters. Selection of PWMs/binding sites was based on the experience we gained on histone promoter binding sites in our previous analysis of histone promoters [24]. The binding sites we used were, TATA-box, CAAT-box, GC-box, E2F binding site, ATF/CREB binding site, Octamer1-box, AC-box, H4TF2 binding site, RT1-box, and TG-box. The PWMs of these boxes and binding sites were compiled by merging non-redundant information gathered from the biological literature, Transfac database [25], and the *ab-initio* motif discovery analysis we conducted earlier [24]. The collected PWMs were then tuned by trial and error for their cutoff threshold parameters on the training on histone promoter sequences; the threshold was set based on whether the PWM was able to identify 90% of the known binding sites in histone promoters. The PWMs with parameters used are shown in the Additional file 2.

### Model building

**Figure 1:**
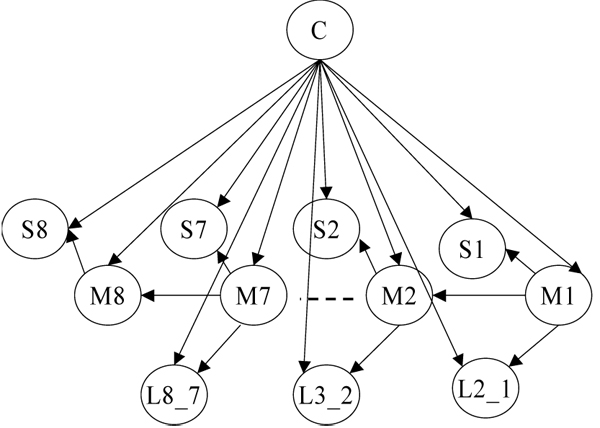
Bayesian network model of histone promoter structure.

Using the selected PWMs we scanned the training sequences with the help of DPM tool and extracted from these sequences our features of interest (motifs, their order of appearance and strand location, and mutual distance between them). All the training sequences contained three motifs or more. We used the extracted features (feature table shown in Additional file 3) to build a Bayesian network model of histone promoter structure, as shown in Figure 1. The model contained 24 nodes including, one Class node and 23 child nodes. The Class node represents the classes of training sequences, and 23 child nodes represent the features associated with each of the motif positions (eight in total) occurring in a training sequence (Mi - motif at position i, and Si - its strand (+/-) for i = 1, .., 8, i increases away from the rightmost end of a sequence, and L(i+1)_i - mutual spacer length between motifs for i = 1, .., 7). A motif position is defined as the relative position of motif occurrence in a sequence with respect to its rightmost (5’) end; thus the first motif that occurs in a sequence from its right end is assigned the first position, similarly the second motif is assigned the second position and so on. The number of motif positions is determined by DPM from the maximum number of motifs present in any training sequence. If no motif occurs in a training sequence for a particular motif position, the associated nodes in the model are characterized by a missing value in the feature table. The model nodes and their states are shown in detail as model definition in Additional file 4. Additional details on the DPM modeling methodology are given in [2].

The promoter model we developed has a predefined structure which reflects the background biology. We relied on the hypothesis that if the promoters of genes have a more conserved order of binding sites with respect to the TSS, their strand location, and mutual distance between them, then there are more chances that genes with such promoters are coregulated. To simplify the model we introduced several assumptions about the dependence relationships between different features in the histone promoters. For example, an augmenting edge between the feature nodes assumes that: i) there is a direct dependence of the first order between adjacent binding sites (i.e. a binding site depends directly only on the preceding binding site), and ii) the mutual distances between the binding sites and their strands depend on the associated binding sites themselves. Each feature node was restricted to have a maximum of one augmenting edge pointing to it. In order to see the effect of dependence relationships between our model features on the performance, we compared our model with a Naïve Bayes model which has no dependence relationships between the features conditional on the Class node.

We estimated the model parameters from the training sequence feature vectors by the EM algorithm [26] using uniform *Dirichlet* parameter priors. The trained model was used to classify a query feature vector to one of the two predefined training sequence classes (histone or background). This was done by assigning a probability value to the query instance for its belonging to one of the two target classes.

### Genome analysis

**Figure 2:**
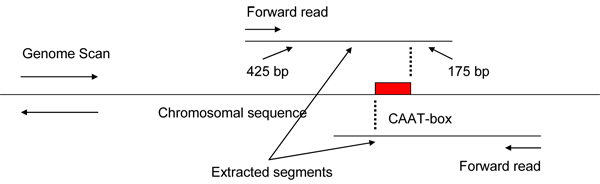
Extraction of segments by scanning the genome using the CAAT-box PWM.

As a preprocessing step, using the ‘*long sequence processing*’ module of DPM, we scanned the entire genome with the PWM of the CAAT-box. This is because CAAT-box was the most frequently occurring pattern in the training histone promoters (60 out of 68 histone promoters). Whenever the CAAT-box was detected on the genome (Figure 2), a segment [-425,+175] with respect to the motif was extracted for further analysis if its GC-nucleotide content was over 37% (which was the minimum value in our training promoter set). The segment coordinates with respect to the CAAT-box were chosen based on the fact that the CAAT-box usually occurs around 75 nucleotides upstream of the TSS in the histone promoters, which means it is located in the proximal promoter region upstream of the TATA-box (~30 nucleotides upstream of TSS) [27]. Biologically, the CAAT-box is a commonly found promoter element that is involved in control of temporal and spatial expression of the associated gene. The genomic segments obtained after the CAAT-box genome scan were all separately scanned with all the 10 PWMs we used in this study. After the PWM scanning, those segments that contained three or more motifs were short listed and fed to the DPM system as query sequences. The DPM system applied the developed histone promoter model to the query sequences, and classified each query sequence to one of the two predefined classes, histone or non-histone. This way, we obtained regions on the genome that DPM predicted as the class of histone promoters.

### Prediction mapping with RefSeq genes

Each of the genomic segment predicted by DPM as histone promoter class was then checked for any gene annotation available for this region using RefSeq human gene data, build HG18, in UCSC Genome Browser. The RefSeq data contained entries for 20,580 unique human genes. The genes whose given TSS was overlapped/covered by the predicted segment on the same strand as the gene in question, were analyzed further.

### Coexpression analysis

We conducted a coexpression analysis on those RefSeq genes whose promoter regions were predicted by our model as ‘histone class’. Based on promoter similarity, we expect that these genes could be coregulated with histone genes. Coexpression could be considered as an indirect, although not necessarily always a correct way, of reflecting coregulation. Coexpression analysis was done to see if we could relate promoter structure similarity to gene expression profile similarity. Typically, we validated two hypotheses in our analysis. First, in general, genes sharing similar promoter structure with histone genes tend to have a higher coexpression level with histone genes compared to those genes that do not share promoter structure with histone genes. Second is in a way a reverse of the first, genes highly coexpressed with histone genes tend to share similar promoter structures with histone genes as well.

To make the coexpression analysis, we downloaded GNF Atlas2 gene expression dataset, which is composed of 79 different human tissues with 2 technical replicates for each tissue, and 33,689 probe sets with 22,283 probe sets contained in Affymetrix HG-U133a platform, and the other 11,406 probe sets designed specially by Affymetrix [28]. The data was first reduced to mono-tissue by taking average of each probe set between two technical replicates. Then, the data was log-transformed and median centralized in each array. Finally, all entries in a single array were normalized to be 1 as a sum-of-squares (see Additional file 5 for downloaded raw data, and Additional file 6 for data after pre-processing). In our GNF expression dataset there were 93 probe sets that belong to histone genes (referred to as *histone probe set*) with their sequences completely contained in histone genes. In addition, there were 1207 probe sets (denoted as *histone coexpression set*), that we found by mapping GeneIDs from RefSeq and chip annotation of GNF Atlas2, to be fully covered by 1,453 genes (denoted as *histone coregulation set*) that we predicted with *histone-like* promoter structure genome-wide (see Results section for details; also Additional file 7 for the lists of probe sets).

### Analysis of Biological terms

For genes that we identified as having similar promoter structure as histone promoters, we tested for enriched Gene Ontology (GO) annotation and protein interaction networks with GOEAST [29] and Ingenuity Pathway Analysis (IPA) system (http://www.ingenuity.com). It was expected that these genes, sharing similar promoter structures with histone genes, would also share with them some molecular functions and biological processes annotation.

## Results

We tested the performance of our histone promoter BN model on the training dataset based on leave-one-out cross-validation. We tested the model on 10 different training sets each with different background sequences and found the performance to be robust with low variability (overall accuracy: 91.3±2.0% with sensitivity: 91.8±2.1%, specificity: 90.7±3.7%, and positive predictive value (ppv)/precision: 90.9±3.2%); the best performance on an individual dataset achieved is: overall accuracy of 93.4%, with sensitivity of 94.1%, specificity of 92.6% and ppv of 92.8% (the best performing dataset is given in Additional file 1). We also found that our model with feature dependencies outperformed a Naive Bayes model which assumes no dependencies between the feature nodes (overall accuracy of Naïve Bayes model was: 88.5±2.1% with sensitivity: 86.9±2.3%, specificity: 90.0±2.7%, and ppv: 89.7±2.6%). We used our best performing model dataset further for the genome scan analysis.

**Figure 3:**
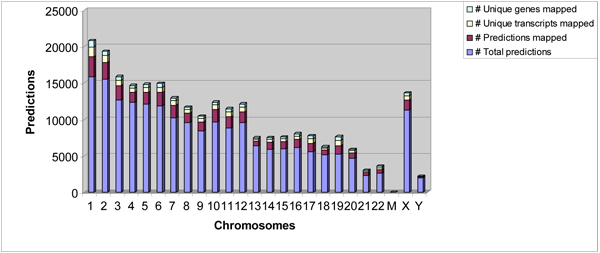
DPM’s 200,180 predictions on different chromosomes and their mappings with known gene transcripts.

**Figure 4:**
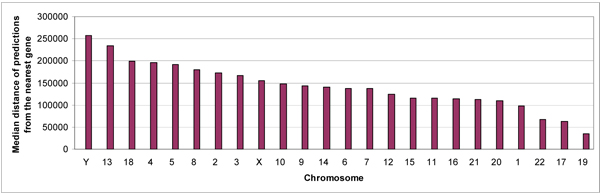
Median chromosomal distance between intergenic predictions and their nearest gene.

In the genome analysis, we extracted 2,710,508 genomic segments based on the CAAT-box predictions by DPM in the initial genome scan. Of these query segments we extracted around the CAAT-box motif, DPM qualified 1,018,182 segments with three motifs or more (refer Additional files 8, 9). Of these, DPM predicted 200,180 as histone class (refer Additional files 10, 11). Of these histone class predictions, 30,611 predictions were associated with 12,899 gene transcripts and their distribution across different chromosomes is shown in Figure 3 (details in Additional file 12). Thus, the majority of the histone-class predictions (169,569) fell in the intergenic regions. The distance between these intergenic predictions with respect to their nearest genes is shown in Figure 4 and varies from a maximum median value in chromosome Y to a minimum value in chromosome 19. We also observed that many of our predictions overlapped with each other. The 200,180 histone class predictions formed 102,670 non-overlapping clusters; equivalent to one histone-class cluster prediction per 29,220 bases.

**Figure 5:**
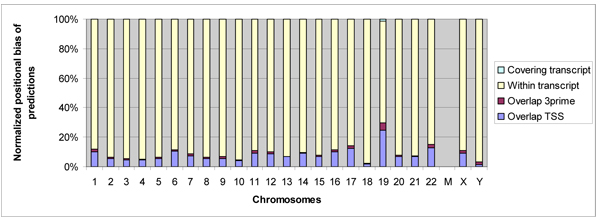
Positional bias of predictions on mapped genes.

**Figure 6:**
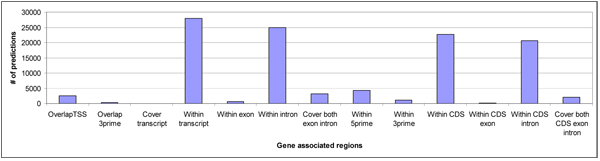
Distribution of predictions on mapped gene regions.

The 12,899 gene transcripts that were associated with DPM predictions correspond to 8,486 known genes. We observed that a large number of predictions that are associated with these known genes fell within their loci, particularly their introns, as can be observed from Figures 5 and 6. This suggests that there could be regulatory regions within the gene loci. Similar observations have previously been reported [30]. Of 8,486 genes that are associated with our predictions, there are 1,453 genes (2,009 transcripts) that form our histone coregulation set, whose promoter regions along with the TSS were covered/mapped by our 2,486 predictions (refer to Additional file 13 for details). These 1,453 genes in the histone coregulation set included 63 histone gene promoters and 1,390 non histone gene promoters that contain the CAAT-box. Of 63 histone promoters, 53 (84%) were part of the training data (refer Table 1), while the remaining ten were from the left-out histone promoters. Thus 77% of 13 histone promoters not used for training were identified by our predictions. In addition, there were two training histone genes (IDs: 94239, 255626) whose TSSs were missed by a narrow margin of 14 and 30 base pairs respectively, though still, we were able to predict a large part of their proximal promoter region. Thus, we were able to correctly recognize a large number of histone promoters from across the genome that contained CAAT-box (63 out of 73 histone genes: 53 out of 60 training histone genes, and 10 out of 13 left-out histone genes).

**Table 1:** Summary of training set and prediction results for histone genes

# of histone genes		predicted in genome scan
total	86	
with CAAT-box	73	63
training set	68	
training set with CAAT-box	60	53
left-out	18	
left-out with CAAT-box	13	10

In our genome analysis, a large number of genomic regions were predicted by DPM as histone class with high probabilities (>0.9). Such regions included, as expected, a large majority of promoters of histone genes (57 of 63) and those of other genes (535 of 1,390); high probability values suggest that the promoters of these genes share certain similarity with histone promoters and these genes thus may have higher likelihood of being coregulated with histone genes.

**Figure 7:**
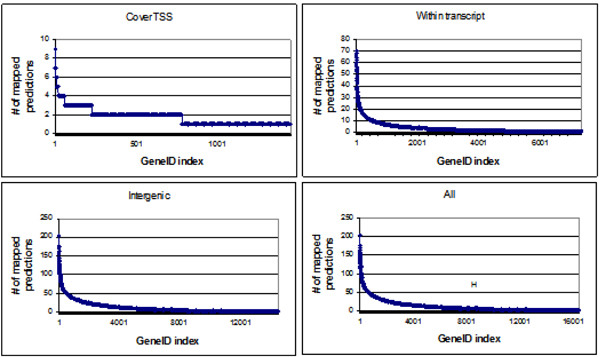
Distribution of prediction mapping with genes. GeneIDs (represented in the graph by their notional indices; refer Additional file 14 to see actual geneIDs) on the left have higher number of mapped predictions.

In order to see how predictions were associated with genes, we plotted the number of our predictions a gene was associated with for the following categories: i) overlap TSS, ii) prediction within transcript, iii) intergenic, and iv) all categories together. For intergenic prediction category, we considered genes closest to the prediction. The results of this analysis are shown in Figure 7. We observed that there were many genes mapped by several predictions in all these categories: the maximum numbers being 9 for overlap TSS, 70 for within transcript, 204 for intergenic, and 204 for all categories together (see Additional file 14 for details). These regions could possibly be related to the regulatory mechanism of the respective genes.

**Figure 8:**
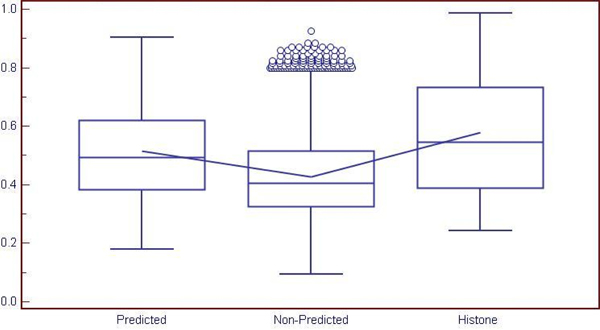
Distribution of CC between Predicted and Non-Predicted probe sets.

To validate the hypothesis that in general genes sharing similar promoter structure with histone genes tend to have a higher coexpression level compared to those that do not share such similarity, we tried to estimate the most significant coexpression with histone probe sets for each probe set in the GNF chip dataset. For this, Pearson’s *centered* correlation coefficient (CC) was calculated between probe sets pairwise. The maximum CC (denoted as maxCC) between a probe set and any of the 93 histone probe sets was then used to indicate the desired coexpression level between that probe set and histone probe sets. Figure 8 shows the distribution of the calculated maxCCs. All probe sets on the chip were classified into two groups based on whether they were covered by the genes predicted as *histone class* or not, which are shown in Figure 8 as *predicted* and *non-predicted*, respectively; there were 1,207 probe sets in the GNF dataset that were covered by 1,453 genes (histone coregulated set) predicted as histone class. The mean values of the two populations were 0.516, with 95% confidence interval (CI) from 0.506 to 0.526, and 0.427, with 95% CI from 0.426 to 0.428, respectively. The t-test score of the difference between the mean of the two populations was -17.164, with p-value<0.0001, which suggests that the predicted group had a coexpression profile significantly different (in our case higher) compared to the non-histone-predicted group even under a very strict statistical significance control. Figure 8 also shows the box-plot for maxCC calculated for each histone probe with respect to the other probes in the histone group (mean value being 0.58, with 95% CI from 0.54 to 0.62), which indicates that the mean of histone group is higher than the predicted group which is expected since histone genes are generally known to express in a fairly synchronized manner.

To validate the converse hypothesis that genes highly coexpressed with histone genes also tend to share some similarity in promoter structure with histone genes, we performed a random test on highly coexpressed genes. 10000 groups of 94 probe sets were randomly selected. For each group, 93 probe sets were treated as histone probe sets and the remaining one was treated as a test probe set to estimate the background of maxCC. The previously calculated maxCC values for probes were then converted into p-values by comparing with the background. These p-values were further converted into the false discovery rate (FDR) [31]. Using a threshold of 0.05 on FDR, we found that there were 141 highly co-expressed probe sets. Out of these probe sets, 65 shared similar promoter structure as histone probe sets while 76 did not. Based on the hypergeometric distribution, we found that the enrichment in genes with promoters similar to histone genes is statistically significant (p-value of 2.12E-55, and corrected p-value for multiplicity testing of 7.13E-51; with hypergeometric parameters of N=33,596, n=141, K=1,207, k=65), thereby validating our hypothesis.

**Figure 9:**
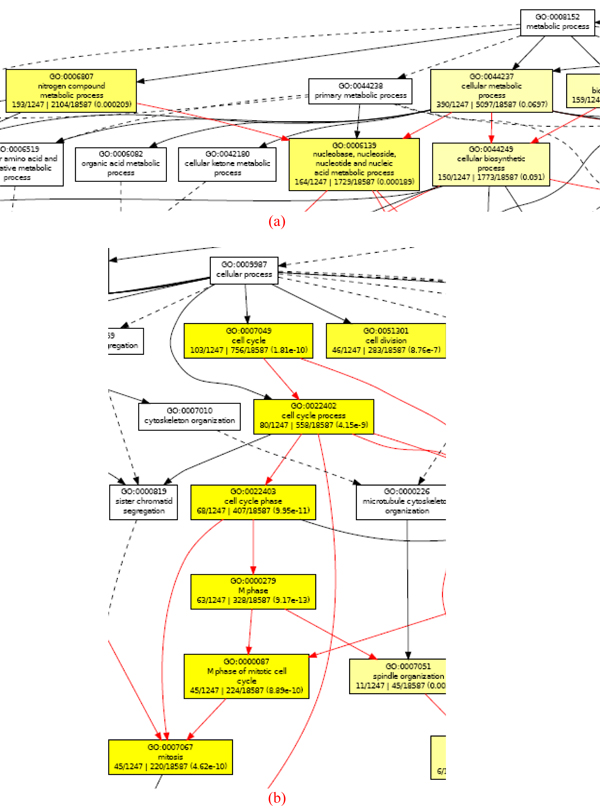
(a) Enriched GO term branch of nucleotide metabolism; (b) Enriched GO term branch of cell cycle regulation.

Results of the biological term analysis conducted on our putative/predicted list of 1,453 putative histone-coregulated genes are shown in Table 2, which lists the top-10 GO terms returned by GOEAST in the three large GO categories, namely biological process, cellular component and molecular function (see Additional files 15, 16, 17, 18 for details). As expected, biological processes and molecular functions requiring histone activity, including nucleosome assembly (0006334), nucleosome organization (0034728), DNA packaging (0006323), chromatin assembly (0031497), DNA binding (0003677), and nucleic acid binding (0003676), are highly ranked. This suggests that many of our putative histone-coregulated genes also co-function or participate in the same processes as histone genes. The metabolic regulation of nucleotide is another branch of terms with statistical significance (Figure 9a). The process of cell cycle regulation is also enriched (Figure 9b), which is also expected, since histone regulation plays a critical role in cell proliferation [1].

**Table 2:** Top GO terms enriched by GOEAST.

GO ID	GO TERM	P-Value
	**Biological Process**	

GO:0006334	nucleosome assembly	3.81E-27
GO:0034728	nucleosome organization	5.08E-27
GO:0006323	DNA packaging	8.63E-27
GO:0031497	chromatin assembly	2.57E-26
GO:0065004	protein-DNA complex assembly	2.82E-25
GO:0006333	chromatin assembly or disassembly	9.14E-25
GO:0006996	organelle organization	3.86E-17
GO:0034621	cellular macromolecular complex subunit organization	2.01E-16
GO:0006325	chromatin organization	2.14E-15
GO:0034622	cellular macromolecular complex assembly	4.93E-15
		

	**Cellular Component**	

GO:0043229	intracellular organelle	9.23E-38
GO:0043227	membrane-bounded organelle	1.75E-37
GO:0043226	organelle	1.79E-37
GO:0043231	intracellular membrane-bounded organelle	2.76E-37
GO:0044424	intracellular part	1.32E-35
GO:0005622	intracellular	4.57E-34
GO:0005634	nucleus	2.81E-33
GO:0000786	nucleosome	5.46E-31
GO:0032993	protein-DNA complex	1.40E-29
GO:0044446	intracellular organelle part	5.47E-24
		

	**Molecular Function**	

GO:0003677	DNA binding	6.43E-25
GO:0003676	nucleic acid binding	7.34E-24
GO:0005488	binding	2.39E-08
GO:0015093	ferrous iron transmembrane transporter activity	1.13E-05
GO:0030528	transcription regulator activity	9.42E-05
GO:0005515	protein binding	6.41E-04
GO:0003747	translation release factor activity	9.13E-04
GO:0008079	translation termination factor activity	9.13E-04
GO:0003690	double-stranded DNA binding	1.07E-03
GO:0043566	structure-specific DNA binding	1.55E-03

**Figure 10:**
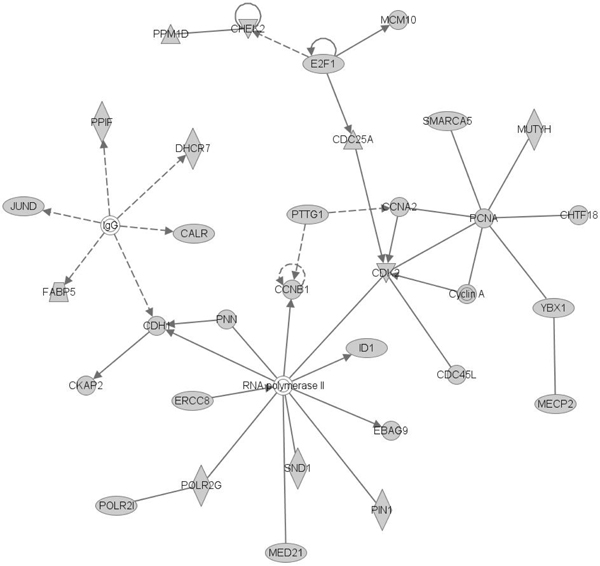
Top ranked network enriched by the predicted gene list with IPA.

**Figure 11:**
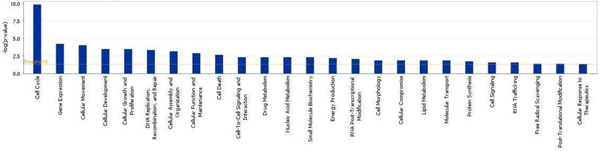
Enriched functions with IPA.

Ingenuity Pathway Analysis (IPA) analysis returns a list of closely associated networks, as well as molecular and cellular functions with the predicted genes. The top ranked networks include cell cycle, DNA replication, gene expression, cellular assembly and organization, and tissue development (see Additional file 19 for details). Figure 10 shows the top ranked network. It involves for example essential cell cycle regulator, CDK2, and cyclins A and B, central DNA replication regulator, RNA polymerase II, and central DNA synthesis regulator E2F. The significantly enriched functions by IPA includes, cell cycle, gene expression, cellular movement, cellular growth and proliferation and DNA replication (Figure 11), which is consistent with the result of GO term enrichment, and further support our hypothesis that genes with similar promoter structure as histone genes tend to coregulate with them.

## Discussion and conclusion

In this study, we developed a computational promoter structure model of human histone genes, an important class of genes that play a crucial role in various cellular processes, such as gene transcription, regulation, chromosome condensation, recombination and replication [1]. The model we developed performed as per our expectation and gave good cross-validation prediction accuracy. In addition, the model was also able to identify a large number of regions across the human genome that have similar structure as histone promoters. These regions include most of our training and left-out histone gene promoters, and suggest 1390 (1453 all predicted gene promoters less 63 histone gene promoters) unique promoters whose genes are likely to be coregulated with histone genes.

Our genome analysis also revealed that a large number of the intragenic predictions (which all had three motifs or more within a span of 600 nucleotides) mapped with the introns located between the coding exons (Figure 6). This suggests that intronic regions might have regulatory function for these genes. Although not validated, these regions may be worth exploring further. Similar observations have also been reported before [30]. In addition, we found that there are some genes associated with a large number of predictions (Figure 7). This bias suggests a more complex regulatory mechanism of these genes. We also observed that there were no predictions on chromosome M. This suggests that the mitochondrial genome contains no gene coregulated with the histone genes. This is in concordance with the fact that mitochondrial genome is free of histones [32] and does not pack into chromatin, which is in contrast to nuclear chromosomes that pack into chromatin with the help of histones.

Our expression analysis demonstrates that genes whose promoter structures were similar to those of histone genes are likely to be ‘more’ coregulated/coexpressed (higher correlation) with them compared to those genes that did not share that similarity (p-value<0.0001). We also found that our pool of 1,453 genes, predicted to be coregulated with histone genes, contained statistically significant number of genes that also coexpressed with histone genes with high correlation. Histone genes are known to have a widespread expression in tissues, like housekeeping genes, both in developmental and differentiated cell-lines [1,9]. This widespread expression pattern of histone genes may be due to the fact that histone proteins play a critical role in variety of chromosomal processes [1], suggesting many genes that are coregulated with histone genes. Overall, our set of 1,453 genes represents possible candidate histone-coregulated genes. We need to emphasize here that coexpression is considered an indirect, though not necessarily completely correct way, of reflecting coregulation.

Our biological term analysis reveals that several genes from our putative list of 1,390 histone coregulated genes share many biological annotation terms amongst themselves. Many of these terms are known to be associated with the histone activity description, indicating that these predicted genes may also have biological function and processes related to histone genes. Thus, there appears a relationship between promoter structure similarity and biological annotation term similarity. This suggests that many of these genes could possibly share similar regulatory mechanism/biological behavior which is also indicative from our coexpression analysis.

In our analysis, we also found that a large majority of histone genes (73 of 86) contain CAAT-box in their promoters, which suggests that histone genes have a strong regulatory and functional relationship with the general transcription factor *NF-Y* that binds to CAAT-box. It is interesting to note that* NF-Y* is a trimer complex comprising YA and two histone H2A-H2B* like* YB-YC subunits [33], and that *NF-Y* is known to have a close constitutive association with core histone proteins of the chromatin complex and can sometimes substitute some of them for functionality [34]. *NF-Y* is also known to play a key role in the transcription regulation of a large number of genes and is believed to activate about 25% of eukaryotic genes [33].

Functional CAAT-box core (i.e. CCAAT) is known to be extremely conserved in the human genome [33]. In our genome-wide scan, we identified 1,355,254 perfect match ‘CCAAT’ sites; this is equivalent to 4,500 nucleotides per prediction. Based on our histone class predictions, we estimate that there could be about 200,000 putative CAAT-box sites in the genome equivalent to one prediction per about 31,000 nucleotides. We also observed that the frequency of the CAAT-box predictions per nucleotide in genes and their promoter regions was nearly six times higher than that in the intergenic regions (assuming 3% of human genome comprises genes and their promoters). This significant bias of the CAAT-box in different genomic regulatory regions has previously been observed [33]. Most of the CAAT-box sites in the genome still remain uncharacterized [35] and this study provides candidates that could be used by researchers for experimental verification.

Human genome remains largely uncharacterized even today, particularly with regard to annotation of regulatory regions and their functions. The reason for this may be attributed to the complexity of the problem. Our study is an attempt to characterize the human genome in the context of histone gene regulation. The methodology that we have demonstrated in this study with CAAT-box can potentially be used to analyze regulatory regions that are associated with other target general transcription factors that may have wide spread activity. Our analysis has resulted in a freely accessible dataset of putative genomic regulatory regions with specific similarity in structure to histone promoters, which we believe is worth exploring further. Apart from being possible false cases, these regions may in part represent regulatory regions (such as promoters, enhancers, silencers and others) associated with genes that are both known and that are possibly yet to be discovered. Further investigations are required for validating functionality of these regions.

## Competing interests

The authors declare that they have no competing interests.

## Authors' contributions

RC and VBB designed, analyzed and implemented the study, performed the experiments and wrote the manuscript. DD contributed to the methodology development, analysis and writing the manuscript. LW and JSL analyzed the results and revised the manuscript. All authors have read and approved the final version of the manuscript.

## Supplementary Material

Additional file 1 Click here for file

Additional file 2 Click here for file

Additional file 3 Click here for file

Additional file 4 Click here for file

Additional file 5 Click here for file

Additional file 6 Click here for file

Additional file 7 Click here for file

Additional file 8 Click here for file

Additional file 9 Click here for file

Additional file 10 Click here for file

Additional file 11 Click here for file

Additional file 12 Click here for file

Additional file 13 Click here for file

Additional file 14 Click here for file

Additional file 15 Click here for file

Additional file 16 Click here for file

Additional file 17 Click here for file

Additional file 18 Click here for file

Additional file 19 Click here for file
