# Supplementary figures and images for: Genome-wide analysis of regions similar to promoters of histone genes
Source: BMC Syst Biol. 2010 May 28;4(Suppl 1):S4. doi: 10.1186/1752-0509-4-S1-S4 (PMC2880410; doi:10.1186/1752-0509-4-S1-S4)

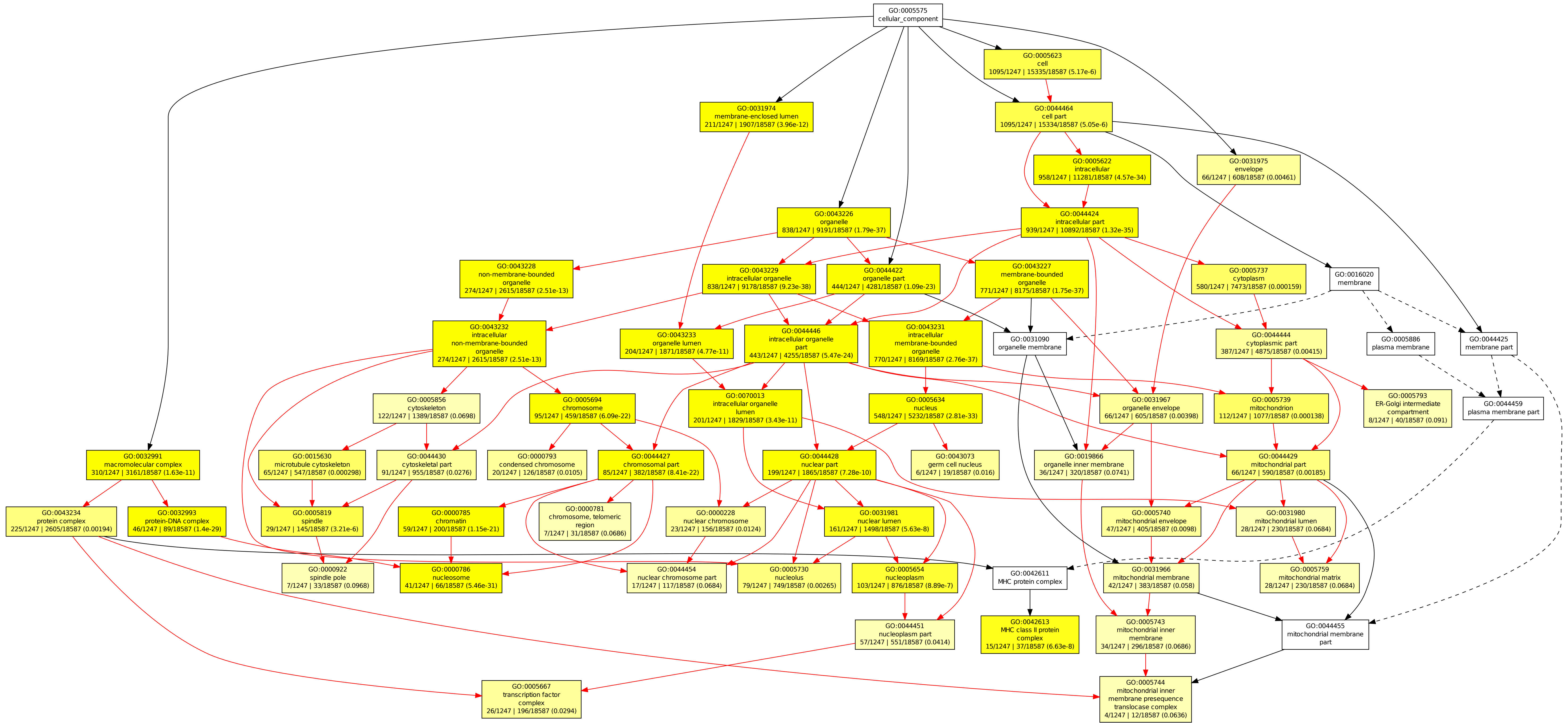

Supplement: Additional file 15 [file 1752-0509-4-S1-S4-S15.pdf]

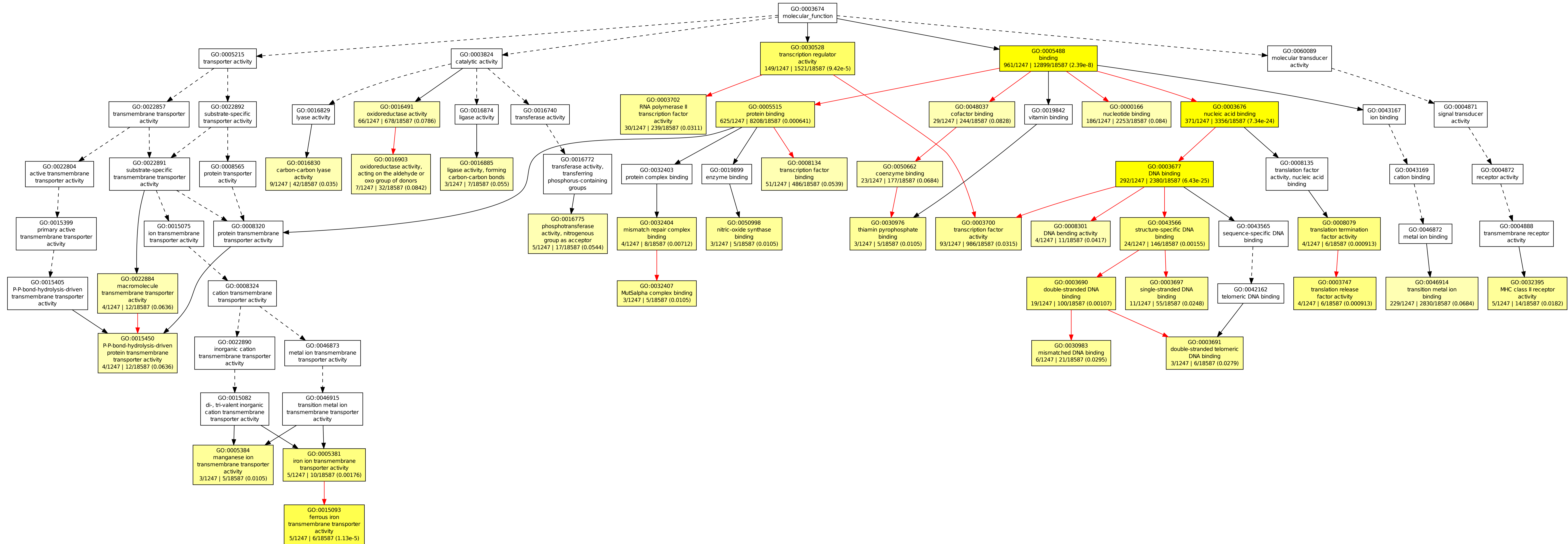

Supplement: Additional file 16 [file 1752-0509-4-S1-S4-S16.pdf]
